# Regulated cell death in chronic kidney disease: current evidence and future clinical perspectives

**DOI:** 10.3389/fcell.2024.1497460

**Published:** 2024-10-31

**Authors:** Kurt T. K. Giuliani, Benjamin C. Adams, Helen G. Healy, Andrew J. Kassianos

**Affiliations:** ^1^ Conjoint Internal Medicine Laboratory, Chemical Pathology, Pathology Queensland, Brisbane, QLD, Australia; ^2^ Kidney Health Service, Royal Brisbane and Women’s Hospital, Brisbane, QLD, Australia; ^3^ Faculty of Medicine, University of Queensland, Brisbane, QLD, Australia

**Keywords:** regulated cell death, hypoxia, chronic inflammation, interstitial fibrosis, chronic kidney disease

## Abstract

Chronic kidney disease (CKD) is the progressive loss of kidney function/structure over a period of at least 3 months. It is characterised histologically by the triad of cell loss, inflammation and fibrosis. This literature review focuses on the forms of cell death that trigger downstream inflammation and fibrosis, collectively called regulated cell death (RCD) pathways. Discrete forms of RCD have emerged as central mediators of CKD pathology. In particular, pathways of regulated necrosis – including mitochondrial permeability transition pore (mPTP)-mediated necrosis, necroptosis, ferroptosis and pyroptosis – have been shown to mediate kidney pathology directly or through the release of danger signals that trigger a pro-inflammatory response, further amplifying tissue injury in a cellular process called necroinflammation. Despite accumulating evidence in pre-clinical models, no clinical studies have yet targeted these RCD modes in human CKD. The review summarizes recent advances in our understanding of RCD pathways in CKD, looks at inter-relations between the pathways (with the emphasis on propagation of death signals) and the evidence for therapeutic targeting of molecules in the RCD pathways to prevent or treat CKD.

## Introduction

Chronic kidney disease (CKD) is a worldwide public health burden characterised by a progressive decline in kidney function. The burden of CKD continues to rise, with global prevalence increasing by ∼25% in the decade 2007-2017 (Xie et al., 2018). The trends underscore fundamental knowledge gaps, starting with the ill-defined pathophysiology of this complex disease. Its pathophysiology is a multi-hit model, with an initial insult triggering pathways of tubular loss/death that activate further hits in inflammatory and fibrosis pathways and, if not switched off, progression to kidney failure (KF) (Yu and Bonventre, 2020; Anders, 2014; Ferenbach and Bonventre, 2015). Newer drugs that slow the progression of CKD have reduced residual risk of KF but by how much is not yet certain - not all people respond to these drugs and none of these agents cure CKD. Innovative strategies that focus on effective targeted treatment of CKD remain a health priority. Here, we review current knowledge of the key cell death pathways in the pathogenesis of CKD, relating functional evidence from studies of experimental animal models to observations made in humans and identifying potential novel therapeutic targets for future clinical management of CKD.

## Chronic inflammation and interstitial fibrosis

Multiple genetic and environmental factors program the kidneys for CKD, including advancing age, uncontrolled hypertension, ethnicity, diabetes, obesity and prior episodes of acute kidney injury (AKI) (Kazancioğlu, 2011). CKD is defined as “*abnormalities of kidney structure or function, present for a minimum of 3 months*” (KDIGO, 2024). Morphologically, CKD is characterised by the histological triad of loss of specialised kidney cells with infiltration of inflammatory cells and fibrosis within the tubulointerstitial compartment, the interstitial tissue adjoining the kidney tubules (Anders, 2014; Haase, 2015; Venkatachalam et al., 2015; Rockey et al., 2015; Nangaku, 2006). The initial insult that causes kidney cell loss/death is usually time-limited. However, where there is a legacy of hypoxia in the environmental niches of these cells, their signalling skews towards pro-inflammatory and fibrotic pathways and further propagation of kidney cell death (Kawakami et al., 2014). Kidney proximal tubular epithelial cells (PTECs) are particularly susceptible to hypoxic dysfunction and cell death in CKD because they rely on mitochondrial fatty acid oxidation as a preferred energy source (Kang et al., 2015). Identifying the specific kidney cell population/s and their regulated cell death (RCD) pathway/s in the inflammatory/fibrotic tubulointerstitium are essential pre-steps in identifying new classes of therapeutics targeting the cell death pathways driving progression of CKD.

## Regulated cell death in CKD

Early descriptions of RCD date from the mid-19th century (Clarke and Clarke, 1996). These early studies were the first to describe the “blebbing” of dying cells which are now termed apoptotic bodies (Clarke and Clarke, 1996). The term “apoptosis” to describe RCD was first introduced by Kerr, Wyllie and Currie in their seminal 1972 paper (Kerr et al., 1972). The field has grown rapidly with distinct molecular processes and multiple pathways of RCD identified {reviewed by [(Tang et al., 2019), (Galluzzi et al., 2018a)]}.

**FIGURE 1:**
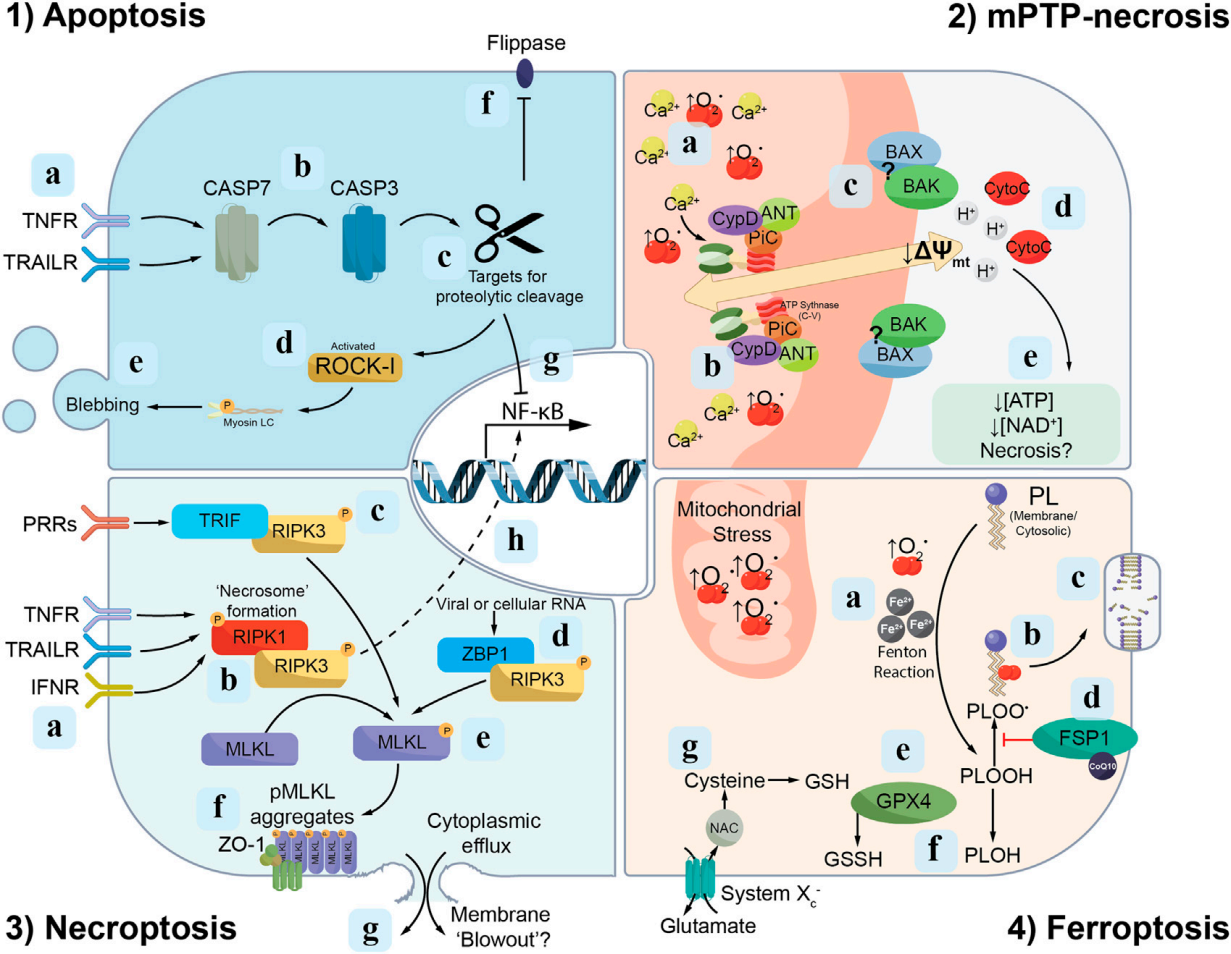
Signalling cascades of discrete modes of RCD. Apoptosis: (1a) The extrinsic apoptosis pathway is initiated following activation of death ligands [e.g., tumour necrosis factor receptor (TNFR) or TNF-related apoptosis-inducing ligand receptors (TRAILR)] by their respective ligands, which in turn triggers (1b) proteolytic cleavage of cysteine proteases, named caspases, culminating in the maturation of caspase-7 (CASP7) and caspase-3 (CASP3). (1c) Active caspase-3 proteolytically cleaves a range of targets, including (1d) ROCK1 which triggers membrane “blebbing” (1e) and apoptotic cell death. (1f) Additionally, active caspase-3 mediates the cleavage of “flippases”, thus permitting phosphatidylserine presentation on the cell membrane outer leaf. (1g) Active caspase-3 also interferes with anti-apoptotic signalling through cleavage of the p65 subunit of nuclear factor-κB (NF-κB). Mitochondrial permeability transition pore (mPTP)-necrosis: (2a) An accumulation of mitochondrial reactive oxygen species (ROS; e.g., ↑O_2_^·^) and calcium (Ca^2+^) trigger the opening of the mPTP. (2b) The F_1_F_0_ subunit of ATP synthase forms part of the inner mitochondrial membrane pore as a component of a “synthasome” complex, consisting of PPIF/cyclophilin D (CypD), ANT and PiC. (2c) The BAX and BAK proteins which accumulate in regions surrounding the mPTP are hypothesised to form part of the outer pore on the cytosolic leaf of the mitochondrial membrane. (2d) Once formed, the mPTP facilitates the release of protons (H^+^) and cytochrome c (CytoC) from the mitochondrial matrix into the cell. Proton release depolarizes the mitochondrial membrane (*↓*∆Ψ_*mt*_), preventing further ATP and NAD^+^ biosynthesis. (2e) The combination of these leads to failed homeostasis and necrotic cell death. Necroptosis: (3a) Activation of TNFR, TRAILR and/or type I interferon receptors (IFNR) mediate the phosphorylation of RIPK-1 and -3, which in turn accumulate into structures called “necrosomes” (3b). (3c-d) Alternative pathways of RIPK3 activation are also identified, including binding to TRIF in response to pattern recognition receptor (PRR) engagement and Z-DNA-binding protein 1 (ZBP1) activated by viral or cellular RNA. (3e) Phosphorylated-RIPK3, in turn, mediates the phosphorylation of MLKL (pMLKL). (3f) pMLKL accumulates on the inner leaf of the cell membrane near Zonula Occludens-1 (ZO-1). (3g) These “hotspots” of pMLKL are hypothesised to overcome a physical membranolytic threshold, which causes membrane blow-out and the lytic release of the cytosol to the extracellular space. (3h) Phosphorylated RIPK3 also interacts with NF-κB signalling components to drive pro-inflammatory cytokine release. Ferroptosis: (4a) The accumulation of free iron (Fe^2+^) and ROS (e.g., from the mitochondria) drives the oxidation of phospholipids and the formation of phospholipid peroxyl radicals (PLOO^·^) (4b). These lipid peroxyl radicals propagate the oxidation of additional phospholipids, which, if not terminated or repaired, will ultimately cause the loss of cell membrane integrity (4c). Repair of oxidised phospholipids may occur via two pathways: the first (4d) involves ferroptosis suppressor protein 1 (FSP1) converting phospholipid peroxyl radicals (PLOO^·^) to phospholipid hydroperoxides (PLOOH). (4e) The second pathway of lipid repair involves glutathione peroxidase 4 (GPX4) reducing in a glutathione (GSH)-dependent reaction phospholipid hydroperoxides (PLOOH) to phospholipid alcohols (PLOH) (4f). (4g) System Xc^−^ consists of a heavy chain component, 4F2hc (SLC3A2) and a transport module, xCT (SLC7A11), and functions as a dedicated cysteine import system, which is an important precursor of glutathione.

**FIGURE 2:**
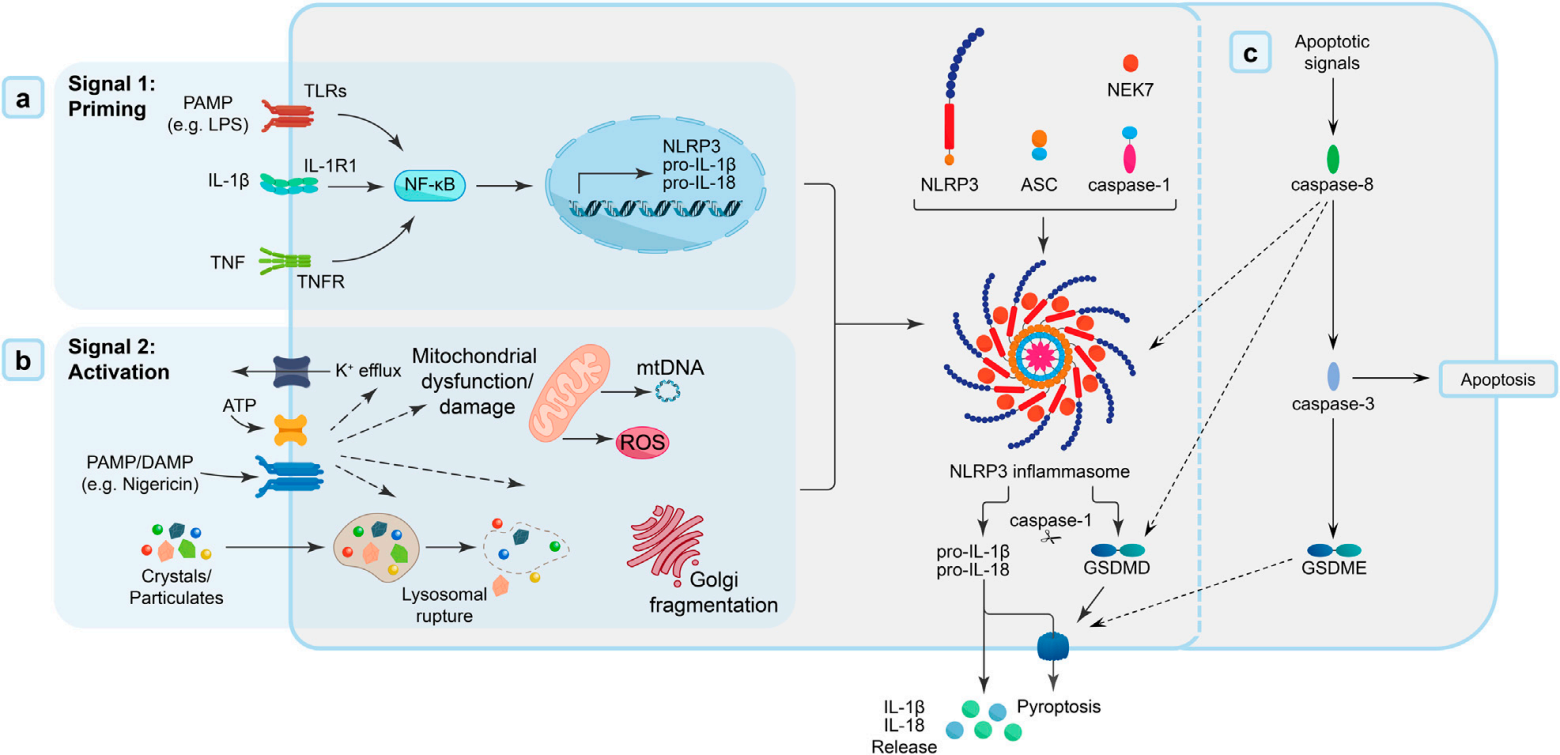
NLRP3 inflammasome priming and activation. **(A)** Signal 1: Priming - Activation of membrane-bound and cytosolic pattern recognition receptors [toll-like receptors (TLRs) by DAMPs/PAMPs (e.g., lipopolysaccharide; LPS), interleukin-1 receptors (IL-1R) by IL-1β, or tumour necrosis factor (TNF) receptors by TNF cytokines] triggers a nuclear factor-κB (NF-κB) signalling cascade, which promotes the transcription of inflammasome components, including NLRP3 and the pro-inflammatory IL-1 family cytokines, IL-1β and IL-18. **(B)** Signal 2: Activation - PAMP and/or DAMP [e.g., extracellular ATP, pore-forming toxins (nigericin), or particular matter] trigger intracellular pathways (mitochondrial dysfunction, oxidative stress) that “activate” the formation of the NLRP3 inflammasome “disk”, a multimeric oligomeric signalling platform, facilitated by the accessory NIMA-related kinase 7 (NEK7) protein. The adaptor protein ASC [apoptosis-like speck-domain containing a caspase activation and recruitment domain (CARD)] is recruited to the inflammasome, and, in-turn, interacts with caspase-1 through homodimeric interactions. Recruitment of caspase-1 to the inflammasome complex mediates the dimerization, autolytic cleavage and activation of caspase-1. Once activated, caspase-1 cleaves pore-forming protein, gasdermin D (GSDMD), and the proinflammatory cytokines, IL-1β and IL-18. Cleaved-GSDMD and active IL-1β/IL-18 accumulate at the cell membrane, where GSDMD forms pores in the cell membrane that facilitate cell lysis/pyroptosis and IL-1β/IL-18 release. **(C)** Caspase-8 activation can also cause pyroptosis either directly through its recruitment into the inflammasome complex or via its cleavage of GSDMD or indirectly by activation of caspase-3 to cleave gasdermin E (GSDME).

The Nomenclature Committee on Cell Death (NCCD) defines RCD as cell death processes that “*rely on dedicated molecular machinery*” which, importantly, can be “*modulated*” through either intrinsic-cellular processes (e.g., genetic) or extrinsic factors (e.g., pharmacological) (Galluzzi et al., 2018a). The NCCD distinguishes RCD from accidental cell death, which is the exposure of a cell to mechanical, chemical or physical insults that rupture the cell membrane causing a loss of cell integrity (Galluzzi et al., 2018a). Cell death in CKD can be caused by both RCD and accidental cell death (i.e., nephrotoxic) mechanisms. This review focuses on pathways of RCD in CKD: (i) weakly immunogenic apoptotic cell death; and (ii) immunogenic/pro-inflammatory necrotic cell death pathways (summarized in Figures 1, 2).

## Apoptosis

Apoptotic cell death occurs in response to either extrinsic or intrinsic factors {reviewed by [(Tang et al., 2019), (Galluzzi et al., 2018a)]}. Despite differences in extrinsic (e.g., death ligand binding to death receptors) *versus* intrinsic [e.g., B-cell lymphoma (BCL)-2-family proteins] activation pathways, both coalesce at the place of autolytic cleavage of cysteine proteases, named caspases - i.e., caspase-8 (extrinsic) or caspase-9 (intrinsic) (Galluzzi et al., 2018a). Activation of these caspases drives the maturation of both caspase-3 and -7, which trigger the terminal cascade of signals resulting in apoptotic cell death (Walsh et al., 2008; McComb et al., 2019). A component of the active caspase-3/-7 signalling cascade is the cleavage/inactivation of “flippases”, which leads to exposure of phosphatidylserines and phosphatidylethanolamines on the outer cell membrane (Kagan et al., 2002; Nagata et al., 2016; Segawa et al., 2018; Segawa et al., 2014). Exposure of these phospholipids to the extracellular environment tags the cell for autophagy and cell death (Kagan et al., 2002). Caspase-3 activity also triggers the characteristic “blebbing” or apoptotic bodies observed in apoptosis (Sebbagh et al., 2001; Aoki et al., 2020). These apoptotic bodies are classically considered non-inflammatory (Savill and Fadok, 2000; Caruso and Poon, 2018). However, in the presence of cellular stress, apoptotic bodies may contain pro-inflammatory damage-associated molecular patterns (DAMPs), including high mobility group box 1 (HMGB1) (Schiller et al., 2013) and interleukin (IL)-1α (Berda-Haddad et al., 2011). These DAMPs are major drivers of sterile inflammatory responses {reviewed by [(Galluzzi et al., 2018a), (Yang et al., 2020), (Belavgeni et al., 2020)]} and thus, may function as potential mediators of CKD progression.

### Apoptosis in CKD

Caspase-3 is identified as a driver of fibrogenesis and long-term kidney dysfunction in a mouse model of acute ischemia-reperfusion injury (IRI), a model of AKI-to-CKD transition (Lan et al., 2021). Despite these findings, this immunologically silent form of RCD is considered a minor pathobiological player in the inflammatory/fibrotic CKD environment (Pefanis et al., 2019; Chen et al., 2018), with evidence that apoptosis may in fact have a more reparative function by clearing excess proliferative myofibroblasts and tubular cells following kidney injury (Chou et al., 2020; Shimizu and Yamanaka, 1993; Sanz et al., 2023). The primary functional role of apoptosis-related molecules in CKD may, in fact, be mediated through their interactions with other regulated necrosis pathways, with activation of caspase-8 during apoptosis shown to inhibit necroptosis, but also trigger pyroptosis (Pang and Vince, 2023; Oberst et al., 2011) (outlined in more detail in subsequent sections).

## Mitochondrial permeability transition pore (mPTP)-mediated necrosis

The mitochondrial permeability transition pore (mPTP) was first described by Haworth and Hunter in 1979 (Haworth and Hunter, 1979). They showed high levels of calcium trigger a non-specific increase in permeability of the inner mitochondrial membrane. Subsequent molecular studies identified elevated inorganic phosphate (P_i_) and oxidative stress, particularly under ischemic or hypoxic conditions (Assaly et al., 2012), as other triggers of mPTP opening and subsequent cell death (Halestrap et al., 2004; Bauer and Murphy, 2020). The discovery that cyclosporin A (CsA) could block the Ca^2+^-dependent pore of the inner membrane responsible for mitochondrial permeability transition was pivotal in identifying a key mPTP-regulating protein: peptidylprolyl cis-trans isomerase F (PPIF) (also known as cyclophilin D) (Crompton and Costi, 1988). Emerging data show the partially disassembled c-subunit ring of F_1_F_0_-ATP synthase (mitochondrial complex V) is part of the core mPTP on the inner mitochondrial membrane (Alavian et al., 2014; Beutner et al., 2017; Mnatsakanyan et al., 2022). These c-subunit rings associate in “synthasome” complexes consisting of PPIF, adenine nucleotide translocase (ANT), phosphate carrier (PiC) and voltage-dependent anion carrier (VDAC) at the inner membrane side of the mPTP and facilitate mitochondrial membrane depolarization (↓∆Ψmt) and necrotic cell death (Beutner et al., 2017).

### mPTP-mediated necrosis in CKD

Mulay et al. (2019) report that *Ppif* deletion or cyclosporin A treatment reduce tubular injury in a small animal model of oxalate-induced AKI. Using electron microscopy, the authors show that oxalate crystals are phagocytosed by tubular epithelial cells (TECs) and associate with disrupted mitochondria (Mulay et al., 2019). In addition, gene ablation of *Ppif* protects mice in an experimental model of IRI-induced AKI (Devalaraja-Narashimha et al., 2009). In AKI models, mPTP-mediated necrosis is identified as an independent RCD pathway that co-exists with other modes of regulated necrosis (i.e., necroptosis) (Mulay et al., 2019; Linkermann et al., 2013a). Although this pathophysiological concept is yet to be extended to CKD, it highlights the potential clinical importance of combination therapies targeting multiple, distinct pathways of RCD for the treatment of kidney diseases.

mPTP-mediated tubular necrosis is reported in a mouse model of unilateral ureteral obstruction (UUO)-induced inflammation and kidney fibrosis/CKD (Leong et al., 2020). Furthermore, Shah *et al* show that apoliprotein (APOL1) risk variants associated with CKD (termed G1 and G2) induce mPTP-mediated necrosis *in vitro* (Shah et al., 2019). The binding of aggregated APOL1 risk variants to mPTP constituents, F_1_F_0_-ATP synthase and ANT, is shown to activate pore opening, mitochondrial dysfunction and cell death (Shah et al., 2019). Despite encouraging data from these *in vivo* and *in vitro* studies, mPTP-mediated necrosis has yet to be demonstrated in kidney tissue from CKD patients. Pharmacological inhibition of mPTP-mediated necrosis using cyclosporin A has been evaluated in myocardial infarction clinical trials (CIRCUS: NCT01502774; and CYCLE: NCT01650662) - although no improvement in patient outcomes were reported (Hausenloy and Yellon, 2015; Monassier et al., 2016; Ottani et al., 2016). Despite the availability of safe therapeutics, confirmation of the physiological relevance of mPTP-mediated necrosis in human CKD is necessary to justify and guide future clinical targeting.

## Necroptosis

Necroptotic cell death occurs in response to homeostatic perturbations, detected by tumour necrosis factor (TNF) receptors (Siegmund et al., 2016), TNF-related apoptosis-inducing ligand receptors (Jouan-Lanhouet et al., 2012) and type I interferon (IFN) receptors (Brault et al., 2018). Activation of these death receptors located on the cell surface phosphorylates cytoplasmic receptor-interacting serine/threonine-protein kinase (RIPK)-1 and −3 (Laurien et al., 2020; Rodriguez et al., 2016), which self-assemble into functional amyloid signalling complexes termed “necrosomes” (Li et al., 2012; Samson et al., 2020). The formation of RIPK1-RIPK3 complexes occurs under conditions that prevent caspase-8-mediated activation – thus, promoting necroptosis over apoptosis (Pang and Vince, 2023). Alternative non-RIPK1-mediated pathways of RIPK3 activation are also identified, including binding to TIR-domain-containing adaptor-inducing interferon-β (TRIF) following triggering of toll-like receptors (TLRs) (Kaiser et al., 2013) or engagement with Z-DNA-binding protein 1 (ZBP1), a sensor of viral or endogenous Z-nucleic acids (Guerrero-Mauvecin et al., 2024).

Activated RIPK3 subsequently phosphorylates mixed lineage kinase domain-like protein (pMLKL) (Monassier et al., 2016), which then oligomerizes and translocates to the inner surface of the cell membrane (Samson et al., 2020). pMLKL at the cell membrane interacts with phosphatidylserine residues (Zargarian et al., 2017) and, in epithelial cells, Zonula Occludens-1 (ZO-1), a tight-junction protein (Samson et al., 2020). The final molecular events in the necroptosis pathway remain unclear (Galluzzi et al., 2018a; Belavgeni et al., 2020). However, Samson *et al* report that pMLKL accumulates into irregularly-shaped “hot-spots” and propose that these MLKL aggregates may overwhelm the cell’s membranolytic threshold to mediate cell death (Samson et al., 2020).

### Necroptosis in CKD

Necroptotic cell death is reported in experimental AKI models of crystal nephropathy (Mulay et al., 2019; Mulay et al., 2016), IRI (Linkermann et al., 2012) and contrast-induced nephropathy (Linkermann et al., 2013b), with administration of necrostatin-1, a RIPK1 inhibitor (Degterev et al., 2005), limiting tubular pathology and restoring kidney function (Linkermann et al., 2012; Linkermann et al., 2013b; Martin-Sanchez et al., 2018). In human rhabdomyolysis-induced AKI kidney tissue, our group has shown increased pMLKL expression localised to sites of tubular injury and adjacent to tubulointerstitial inflammation (TNF-α) and immune cell infiltration (i.e., macrophages, dendritic cells (DCs) and T lymphocytes) (Grivei et al., 2020).

RIPK3-MLKL-mediated tubular loss and necroinflammation also contribute to CKD progression in rats that have undergone subtotal nephrectomy (Zhu et al., 2016) and AKI-to-CKD transition in a mouse IRI model (Chen et al., 2018). In particular, Chen *et al* (Chen et al., 2018) show gene deletion of *Ripk3* or *Mlkl* ameliorates kidney tubular cell necroptosis, macrophage infiltration/activation and, in the long-term, tubulointerstitial fibrogenesis after IRI. *Ripk3* knockout also reduces kidney fibrosis in mouse models of adenine-induced CKD, UUO and diabetic nephropathy (Imamura et al., 2018; Shi et al., 2020). In human kidney biopsies from CKD patients with diabetic nephropathy and histological evidence of tubulointerstitial fibrosis, RIPK3 expression is increased compared with control kidney tissue (Imamura et al., 2018). Further interrogation of RIPK3 function in CKD is needed, including: (i) the role of the caspase-8/RIPK3 signalling axis in regulating the delicate balance between non-inflammatory apoptosis and pro-inflammatory necroptosis (Xu and Huang, 2022); (ii) ZBP1 function in RIPK3 activation during viral-associated nephropathies; and (iii) how RIPK3 may trigger inflammation, independently of necroptosis – i.e., through activation of nuclear factor-κB (NF-κB) or the inflammasome (Moriwaki et al., 2017).

## Ferroptosis

The NCCD defines ferroptosis as *“a form of RCD initiated by…severe lipid peroxidation, which relies on reactive oxygen species generation and iron availability”* (Galluzzi et al., 2018a). Ferroptotic death occurs when free radicals (e.g., reactive oxygen species; ROS), generated either via the ferrous (Fe^2+^) iron-dependent Fenton reaction or via the mitochondrial respiratory chain, attack and oxidize cell membrane phospholipids (Galluzzi et al., 2018a). The accumulation of toxic lipid peroxides and their breakdown products (e.g., 4-hydroxynonenal (4-HNE) and malondialdehyde (MDA)) disrupt cell membrane integrity, leading to lytic cell death and release of inflammatory DAMPs (Chen et al., 2024). Oxidized phospholipids are repaired through two pathways, with: (i) ferroptosis suppressor protein 1 (FSP1) reducing coenzyme Q_10_, which, in turn, acts as a lipophilic radical-trapping antioxidant to halt the propagation of lipid peroxides (Bersuker et al., 2019); or (ii) glutathione peroxidase 4 (GPX4) reducing phospholipid hydroperoxides to non-toxic phospholipid alcohols using glutathione (GSH) as a co-substrate (Berndt et al., 2024). SLC7A11 (commonly known as xCT) is an important regulator of this second pathway, importing cysteine for glutathione biosynthesis (Koppula et al., 2021). Biomarkers of ferroptotic cell death therefore include reduced expression of FSP1, GPX4 and SLC7A11 (Berndt et al., 2024). Ferroptosis is distinguished biologically from other RCD modes by two features: (i) it lacks a terminal executioner protein (Galluzzi et al., 2018b); and (ii) spreads through organised cell populations in a non-random, wave-like pattern (Linkermann et al., 2014; Kim et al., 2016; Riegman et al., 2020; Katikaneni et al., 2020).

### Ferroptosis in CKD

Ferroptotic tubular cell death has been reported in mouse AKI models of calcium oxalate nephropathy (Linkermann et al., 2014), folic acid-induced AKI (Martin-Sanchez et al., 2017), IRI (Linkermann et al., 2014; Su et al., 2019), cisplatin-induced nephropathy (Deng et al., 2019) and rhabdomyolysis (Guerrero-Hue et al., 2019). In humans, *in situ* immunolabelling of diagnostic biopsies reported as AKI found elevated tubular ferroptosis to be restricted to patients in whom the AKI progressed clinically to CKD (i.e., where patients undergo AKI-to-CKD transition) (Wang et al., 2024).

Kidney tubular ferroptosis has also been demonstrated in murine models of diabetic nephropathy (Kim et al., 2021), UUO-induced kidney fibrosis (Zhang B. et al., 2021) and adenine-induced CKD (Khan et al., 2022), with amelioration of tubular injury/interstitial fibrosis in animals treated with the ferroptosis inhibitors, ferrostatin-1, liproxstatin-1 and XJB-5-131 (Kim et al., 2021; Zhang B. et al., 2021; Zhao et al., 2020). Ferroptosis has been reported in human PTECs (↓GPX4, ↑4-HNE) in an *in vitro* hypoxic model of human CKD and *in situ* within human fibrotic kidney tissue (Giuliani et al., 2022). The Linkermann group proposed successive ferroptotic “waves of death” are the drivers of CKD progression (Belavgeni et al., 2020; Maremonti et al., 2022). Recent publications propose that a ferroptotic ‘wave of tubular death’ may be propagated to neighbouring cells via: (i) cell-cell contacts (Roeck et al., 2023); (ii) an osmotic mechanism independently of cell rupture (Riegman et al., 2020); (iii) small extracellular vesicles (Wang et al., 2024); or (iv) a redox imbalance in the local micro-environment (Belavgeni et al., 2020; Maremonti et al., 2022). The mechanism/s by which ferroptotic cell death is transmitted along the tubular compartment in CKD remains an intense area of research investigation.

## Pyroptosis

In contrast to these previously defined forms of RCD, pyroptosis is strongly associated with the innate immune system - e.g., neutrophils, monocytes/macrophages, DCs (Liu et al., 2023). Pyroptosis is triggered in response to pathogens (i.e., pathogen-associated molecular patterns; PAMPs) and/or tissue damage (i.e., DAMPs), which drive the formation of multi-protein signalling platforms termed “inflammasomes” and result in caspase-1 activation (Monteleone et al., 2018; Broz and Dixit, 2016; Anders, 2016). Active caspase-1 cleaves pro-inflammatory cytokines, IL-1β and IL-18, and pore-forming protein, gasdermin D (GSDMD), into their mature forms (Boucher et al., 2018; Vijayaraj et al., 2021; Liu et al., 2020; Xia et al., 2021). Cleaved GSDMD accumulates on the inner leaf of the cell membrane where it self-oligomerizes into a size and charge exclusive pore, resulting in plasma membrane rupture, cell lysis and the release of the mature IL-1β/IL-18 (Xia et al., 2021; Liu et al., 2016). Further highlighting the complex molecular links between apoptosis and other discrete forms of regulated necrosis, caspase-8 activation can also trigger pyroptosis either directly via its recruitment into the inflammasome complex or through its cleavage of GSDMD or indirectly by activating caspase-3 to cleave gasdermin E (GSDME) (Pang and Vince, 2023). The NLRP3 (NACHT, leucine-rich-repeat (LRR), and pyrin domain (PYD)-containing protein 3) inflammasome is the most extensively characterised of the inflammasome family {reviewed by [(Swanson et al., 2019), (Broz and Dixit, 2016)]}. Canonical NLRP3 inflammasome activation by danger signals (PAMPs and/or DAMPs) involves both priming and activation steps (summarized in Figure 2). NLRP3 inflammasome activation is implicated in an array of inflammatory pathobiologies {reviewed in [(Coll et al., 2022), (Hutton et al., 2016)]}, including Alzheimer’s disease (Saresella et al., 2016; Heneka et al., 2013; Venegas et al., 2017), cryopyrin-associated periodic syndrome (Leslie et al., 2006; Lachmann et al., 2009), type 2 diabetes (Masters et al., 2010; Lei et al., 2019), and both acute and chronic kidney diseases (Lei et al., 2019; Vilaysane et al., 2010; Ludwig-Portugall et al., 2016).

### Pyroptosis in CKD

The pro-fibrotic role of inflammasome activation and downstream pyroptotic cell death is established in experimental CKD models. NLRP3 inflammasome activation is a critical driver of kidney fibrosis in murine models of diabetic nephropathy (Wang MZ. et al., 2022), oxalate nephropathy (Ludwig-Portugall et al., 2016), adenine-induced CKD (Ludwig-Portugall et al., 2016) and UUO (Seo et al., 2019), while *Gsdmd* deletion is shown to alleviate fibrosis in experimental models of obstructive nephropathy (Wang Y. et al., 2022) and APOL1-associated podocytopathy (Wu J. et al., 2021). Inflammasome activation in murine models of kidney fibrosis is predominantly restricted to inflammatory cells (i.e., neutrophils, macrophages, DCs) (Ludwig-Portugall et al., 2016; Wang Y. et al., 2022; Chi et al., 2017). However, evidence in kidney parenchymal cells (i.e., TECs) is also reported (Chi et al., 2017), including a non-canonical pathway of caspase-3/GSDME-mediated pyroptosis in TECs, but not haematopoietic cells, promoting inflammation (i.e., macrophage activation) and fibrosis in mouse UUO models (Li et al., 2021). Notably, deletion of *Gsdme* also attenuates kidney fibrosis after UUO or subtotal nephrectomy (Li et al., 2021; Wu M. et al., 2021). It remains to be established if these alternative pathways of caspase-3 and/or caspase-8-mediated pyroptosis play a dominant pathophysiological role or ‘back-up’ function in CKD.

In humans, the NLRP3 inflammasome has been strongly associated with tubulointerstitial injury/fibrosis and CKD progression (Vilaysane et al., 2010; Ermer et al., 2016; Darisipudi and Knauf, 2016; Shahzad et al., 2015; Anders and Muruve, 2011; Granata et al., 2015). Elevated levels of IL-1β and IL-18 are also reported in human fibrotic kidney tissue (Law et al., 2019), with downstream stimulation of human TECs with IL-1β inducing pathways of oxidative stress and fibrogenesis (Vesey et al., 2002; Vesey et al., 2005). However, the cellular origin of inflammasome activation and pyroptosis in human CKD (i.e., the respective contribution of inflammatory vs. parenchymal cells) is controversial. Our group has identified tubulointerstitial CD1c^+^ DC as a key immunological source of inflammasome activation within human fibrotic kidney tissue (Giuliani et al., 2022). In contrast, while human kidney tubular epithelial cells express components of the inflammasome machinery (Kim et al., 2018), whether they can form active inflammasome complexes and undergo pyroptosis is yet to be unequivocally established. Moreover, pre-clinical studies are required to determine whether gasdermin/pyroptosis inhibitors with demonstrated efficacy in UUO murine models (e.g., disulfiram) (Zhang Y. et al., 2021) are of therapeutic benefit in treating human kidney fibrosis.

## Future directions and conclusions

With an ageing population and increasing global prevalence of diabetes and hypertension, the burden of CKD is predicted to rise to the fifth most common cause of death by 2040 (Foreman et al., 2018). Novel therapeutic strategies that target the pathobiological pathways underpinning the development of tubulointerstitial inflammation and fibrosis are a health priority in CKD management. Accumulating evidence from experimental murine models and human pre-clinical studies identify discrete forms of RCD as central pro-inflammatory/fibrotic drivers of CKD. However, answers to key and unresolved questions in this field of RCD research are still required to support precision targeting in the clinical setting:

1. Are distinct kidney parenchymal cell types more susceptible to a particular mode of RCD in CKD? Our group identify ferroptosis (↓GPX4, ↑4-HNE) as the primary form of RCD in human PTECs under hypoxic CKD conditions, with no evidence of apoptosis (cleaved caspase-3), mPTP-mediated necrosis (PPIF) or necroptosis (pMLKL) (Giuliani et al., 2022). Is this selective induction of a discrete RCD mode in PTECs also applicable to cells upstream (i.e., within the glomerular compartment) and downstream of the kidney proximal tubules (i.e., within other tubular segments - loop of Henle, distal tubules)?
2. Is cross-talk between different RCD pathways important in CKD progression? RCD machinery exhibits surprising flexibility, capable of non-canonical functional roles and triggering cross-talk between different cell death modes {also reviewed by [(Sanz et al., 2023)]}. For example, a convergence of pyroptosis, apoptosis and necroptosis termed PANoptosis has been recently identified, with cross-talk between these individual RCD pathways mediated through the generation of a multi-protein PANoptosome complex that includes caspases, RIPK1, RIPK3 and ZBP1 (Pandian and Kanneganti, 2022). Although PANoptosis of kidney vascular endothelial cells is reported in mice with trichloroethylene-induced AKI (Xie et al., 2024), translation of this work to CKD models is necessary to establish the pathobiological relevance of this complex inflammatory cell death pathway in fibrogenesis.
3. What are the mechanism/s that propagate RCD within the CKD micro-environment? Does transmission of RCD through the kidney occur: (i) in a random manner; (ii) in a synchronised, wave-like form restricted to a specific RCD mode (i.e., a ferroptotic “wave of death”) (Belavgeni et al., 2020; Maremonti et al., 2022); (iii) via a process of necroinflammation, where regulated necrosis triggers an inflammatory response and downstream secondary necrosis (i.e., ferroptotic PTECs induce inflammasome activation/pyroptosis in tubulointerstitial DCs) (Giuliani et al., 2022); or (iv) via a combination of all events.
4. Which RCD inhibitors have clinical potential as CKD therapeutics? Although several inhibitors of RCD have been evaluated for their anti-fibrotic effects in both *in vitro and in vivo* models of CKD, their suitability for clinical applications is unclear. Ferrostatin-1, a potent small-molecule compound that blocks lipid peroxidation (Skouta et al., 2014), is the gold-standard ferroptosis inhibitor in pre-clinical CKD studies (Khan et al., 2022; Giuliani et al., 2022). However, the clinical translatability of ferrostatin-1 is limited by its poor *in vivo* metabolic stability (Devisscher et al., 2018). Similarly, NLRP3 inflammasome inhibitor, MCC950, attenuates kidney fibrosis in mouse models of crystal nephropathy (Ludwig-Portugall et al., 2016). However, a phase II rheumatoid arthritis clinical trial evaluating MCC950 efficacy was suspended due to off-target liver toxicity in patients (Mullard, 2019). Further evaluation of the mechanisms that regulate RCD in fibrotic kidneys will enable the discovery of novel targeted inhibitors or repurposing of established (FDA-approved) drugs for the treatment of patients with CKD.
